# Supplementary material for: Time-Series RNA Sequencing Reveals Temperature-Specific and Temperature-Compensated Circadian Genes in Drosophila
Source: bioRxiv. 2025 Nov 4:2025.11.04.686609. Preprint. [Version 1] doi: 10.1101/2025.11.04.686609 (PMC12637615; doi:10.1101/2025.11.04.686609)
Supplement: 10 [file NIHPP2025.11.04.686609v1-supplement-10.pdf]

bioRxiv preprint doi: https://doi.org/10.1101/2025.11.04.686609; this version posted November 4, 2025. The copyright holder has placed this preprint (which was not certified by peer review) in the Public Domain. It is no longer restricted by copyright. Anyone can legally share, reuse, remix, or adapt this material for any purpose without crediting the original authors.

Tables:

Table S1. Summary of RNA-seq data quality and mapping statistics

File name: TableS1_RNAseq_QC_Mapping.xlsx

Table S2. Oscillating genes identified using JTK_CYCLE

File name: TableS2_JTK_CYCLE.result.xlsx

Table S3. Results of differential gene expression between T18.DD and T25.DD

File name: TableS3_T18.DD_vs_T25.DD.diff.result.xlsx

Table S4. Results of differential gene expression between T29.DD and T25.DD

File name: TableS4_T29.DD_vs_T25.DD.diff.result.xlsx

Table S5. Results of differential gene expression between T25.LD and T25.DD

File name: TableS5_T25.LD_vs_T25.DD.diff.result.xlsx

Table S6. Amplitude comparison of shared oscillating genes under T18.DD and T25.DD

File name: TableS6_T18DD_vs_T25DD.amplitude_comparison_results.xlsx

Table S7. Amplitude comparison of shared oscillating genes under T18.DD and T29.DD

File name: TableS7_T18DD_vs_T29DD.amplitude_comparison_results.xlsx

Table S8. Amplitude comparison of shared oscillating genes under T29.DD and T25.DD

File name: TableS8_T29DD_vs_T25DD.amplitude_comparison_results.xlsx

Table S9. Gene Ontology enrichment analysis of oscillating genes under DD conditions

File name: TableS9_Enrichment_AllLists.xlsx

bioRxiv preprint doi: https://doi.org/10.1101/2025.11.04.686609; this version posted November 4, 2025. The copyright holder has placed this preprint (which was not certified by peer review) in the Public Domain. It is no longer restricted by copyright. Anyone can legally share, reuse, remix, or adapt this material for any purpose without crediting the original authors.

Supplementary Figures

Figure S1

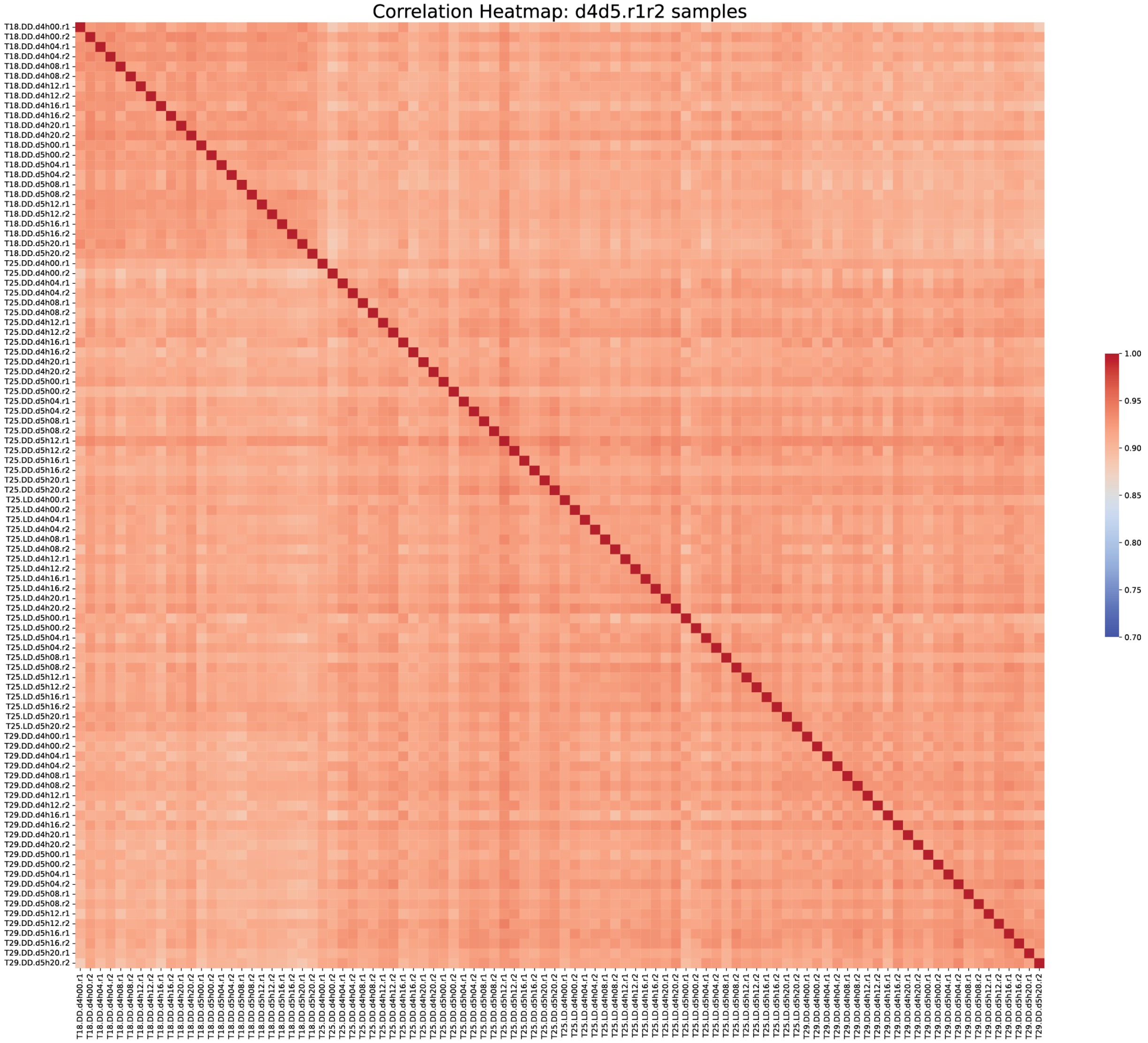

Figure S1. Correlation matrix of the 96 time-series RNA-seq samples.

bioRxiv preprint doi: https://doi.org/10.1101/2025.11.04.686609; this version posted November 4, 2025. The copyright holder has placed this preprint (which was not certified by peer review) in the Public Domain. It is no longer restricted by copyright. Anyone can legally share, reuse, remix, or adapt this material for any purpose without crediting the original authors.

Figure S2

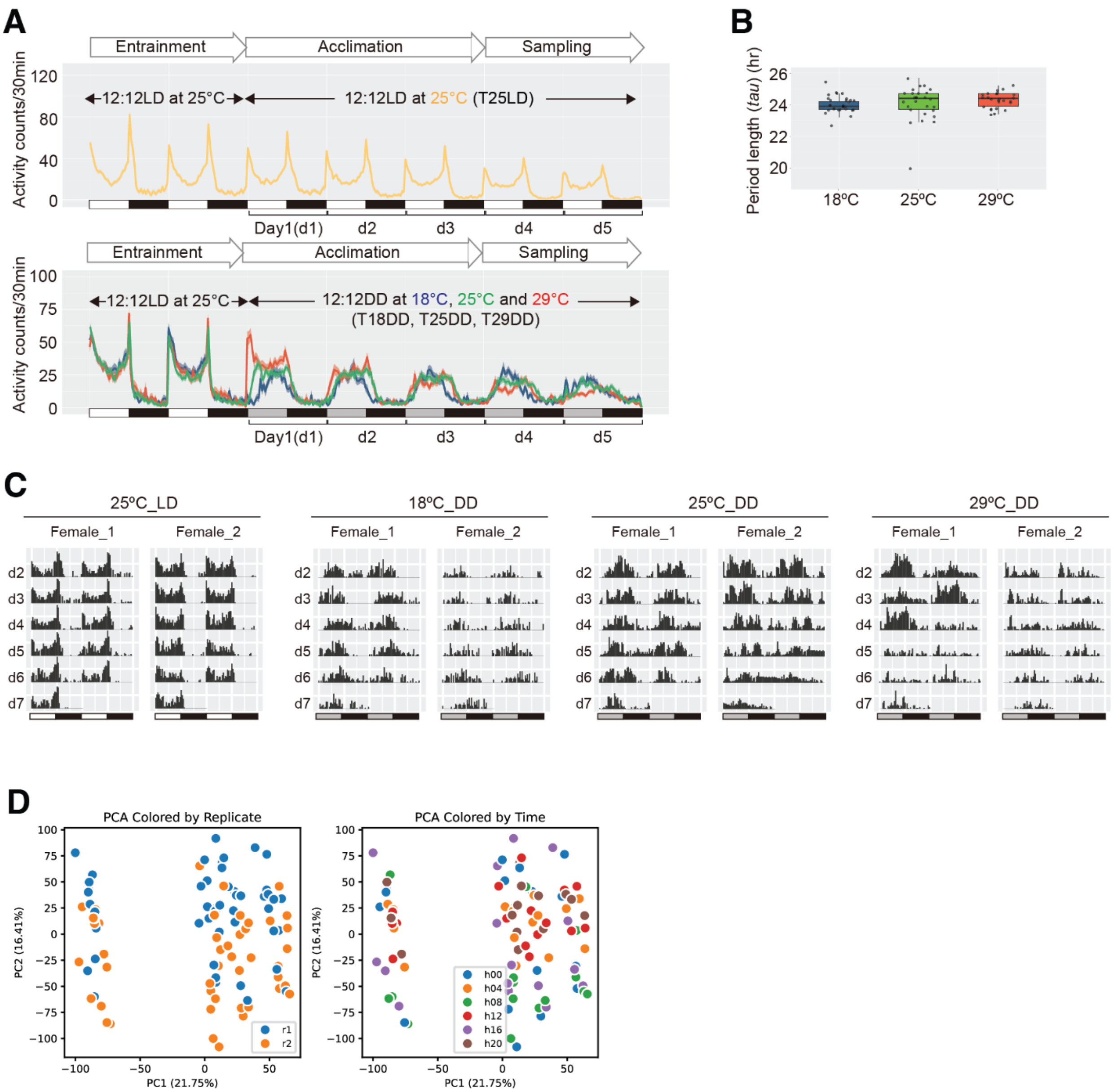

Figure S2. Overview of experiment design for time series RNA sequencing sample collections under different temperature conditions and locomotor activity of female animals under these conditions.

(A) Locomotor activity of female wild-type flies under the 25 °C 12:12 LD condition (upper panel: T25DD, yellow) and three 12:12 DD temperature conditions (lower panel: T18DD, blue; T25DD, green; T29DD, red). Under the T25LD condition (upper panel), flies were monitored at 25 °C LD for two LD cycles (entrainment) before continuing

bioRxiv preprint doi: https://doi.org/10.1101/2025.11.04.686609; this version posted November 4, 2025. The copyright holder has placed this preprint (which was not certified by peer review) in the Public Domain. It is no longer restricted by copyright. Anyone can legally share, reuse, remix, or adapt this material for any purpose without crediting the original authors.

under 25 °C LD (acclimation) for three LD cycles (Day 1–Day 3, d1–d3). Under constant darkness (lower panel), flies were monitored at 25 °C LD for two LD cycles (entrainment) before being transferred to 18 °C DD, 25 °C DD, or 29 °C DD (acclimation) for three DD cycles (d1–d3).

(B) PCA plots of the 96 RNA-seq samples, colored by collection condition (upper panel) or collection day (lower panel).

(C) Representative double plots of locomotor activity of female flies under 25°C LD, 18°C DD, 25°C DD, and 29°C DD conditions.

(D) PCA plots of the 96 RNA-seq samples, colored by batch of replicate (left panel, replicate 1, r1; replicate 2, r2) or timepoint (right panel, h00 includes ZT00, ZT24, CT00, and CT24; h04 includes ZT04, ZT28, CT04, and CT28; h08 includes ZT08, ZT32, CT08, and CT32; h12 includes ZT12, ZT36, CT12, and CT36; h16 includes ZT16, ZT40, CT00, and CT40; h20 includes ZT20, ZT44, CT20, and CT44).

bioRxiv preprint doi: https://doi.org/10.1101/2025.11.04.686609; this version posted November 4, 2025. The copyright holder has placed this preprint (which was not certified by peer review) in the Public Domain. It is no longer restricted by copyright. Anyone can legally share, reuse, remix, or adapt this material for any purpose without crediting the original authors.

Figure S3

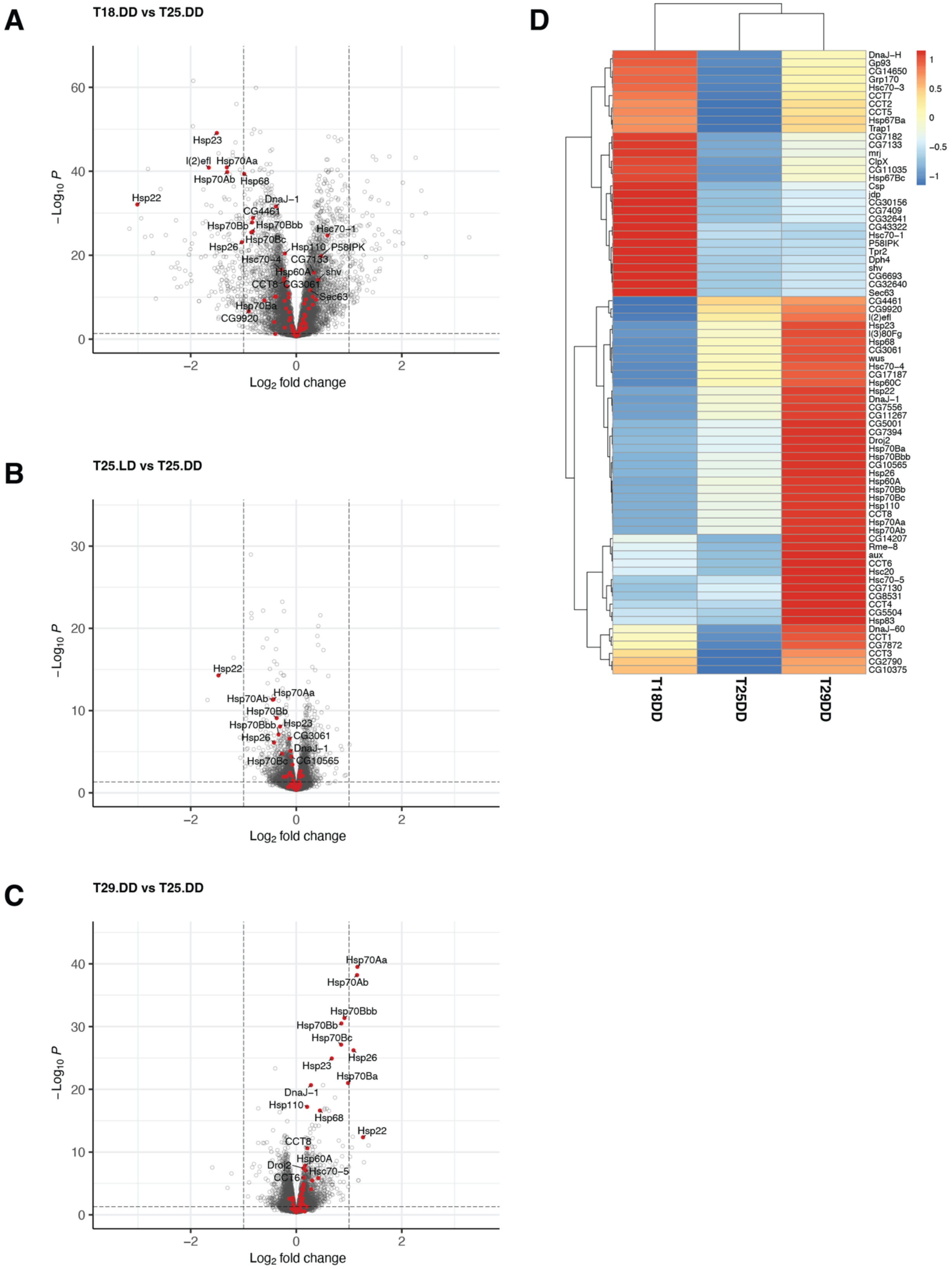

bioRxiv preprint doi: https://doi.org/10.1101/2025.11.04.686609; this version posted November 4, 2025. The copyright holder has placed this preprint (which was not certified by peer review) in the Public Domain. It is no longer restricted by copyright. Anyone can legally share, reuse, remix, or adapt this material for any purpose without crediting the original authors.

Figure S3. Differential gene expression analysis between constant darkness conditions.

(A-C) Volcano plots of differential gene expression analysis. Comparison between 18 °C DD and 25 °C DD conditions (A), comparison between 25 °C LD and 25 °C DD conditions (B), and comparison between 29 °C LD and 25 °C DD conditions. Red dots indicate genes belonging to the gene group of HEAT SHOCK PROTEINS (HSP) (FlyBase ID: FBgg0000501).

(D) Heatmap showing the expression levels of the HSP genes under the three constant darkness conditions.

bioRxiv preprint doi: https://doi.org/10.1101/2025.11.04.686609; this version posted November 4, 2025. The copyright holder has placed this preprint (which was not certified by peer review) in the Public Domain. It is no longer restricted by copyright. Anyone can legally share, reuse, remix, or adapt this material for any purpose without crediting the original authors.

Figure S4

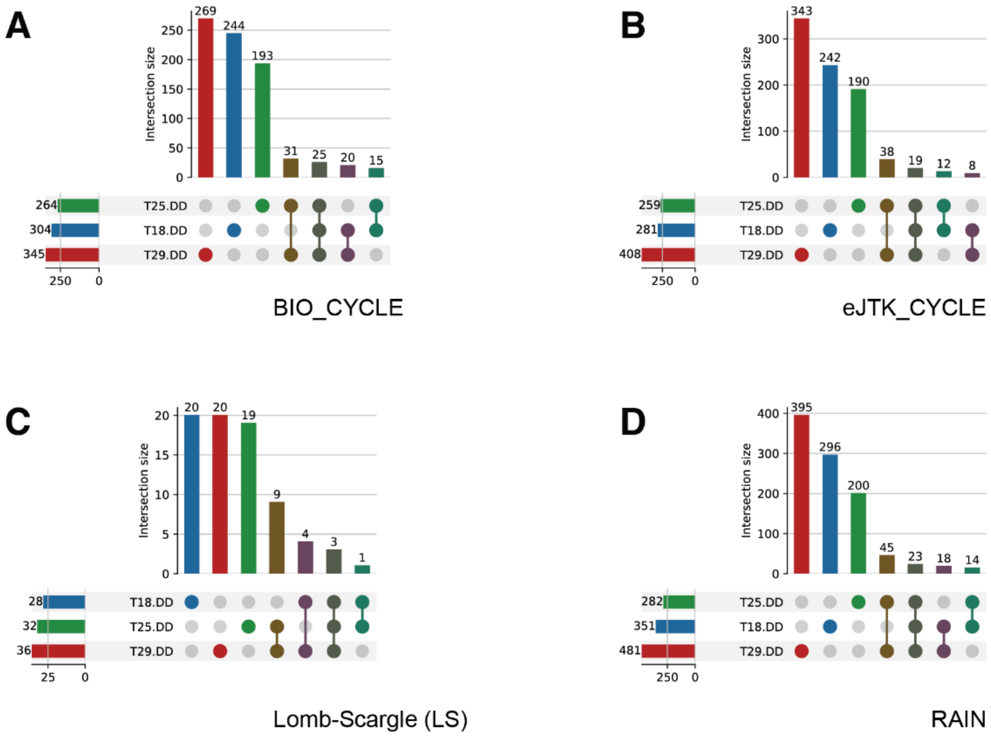

Figure S4. Numbers of oscillating genes identified using additional algorithms. **(A-D)** Upset plots showing numbers of genes that were identified as oscillating genes under one, two, or all three DD conditions, using BIO_CYCLE (A), eJTK_CYCLE (B), Lomb-Scargle (LS) (C) and RAIN (D).

bioRxiv preprint doi: https://doi.org/10.1101/2025.11.04.686609; this version posted November 4, 2025. The copyright holder has placed this preprint (which was not certified by peer review) in the Public Domain. It is no longer restricted by copyright. Anyone can legally share, reuse, remix, or adapt this material for any purpose without crediting the original authors.

Figure S5

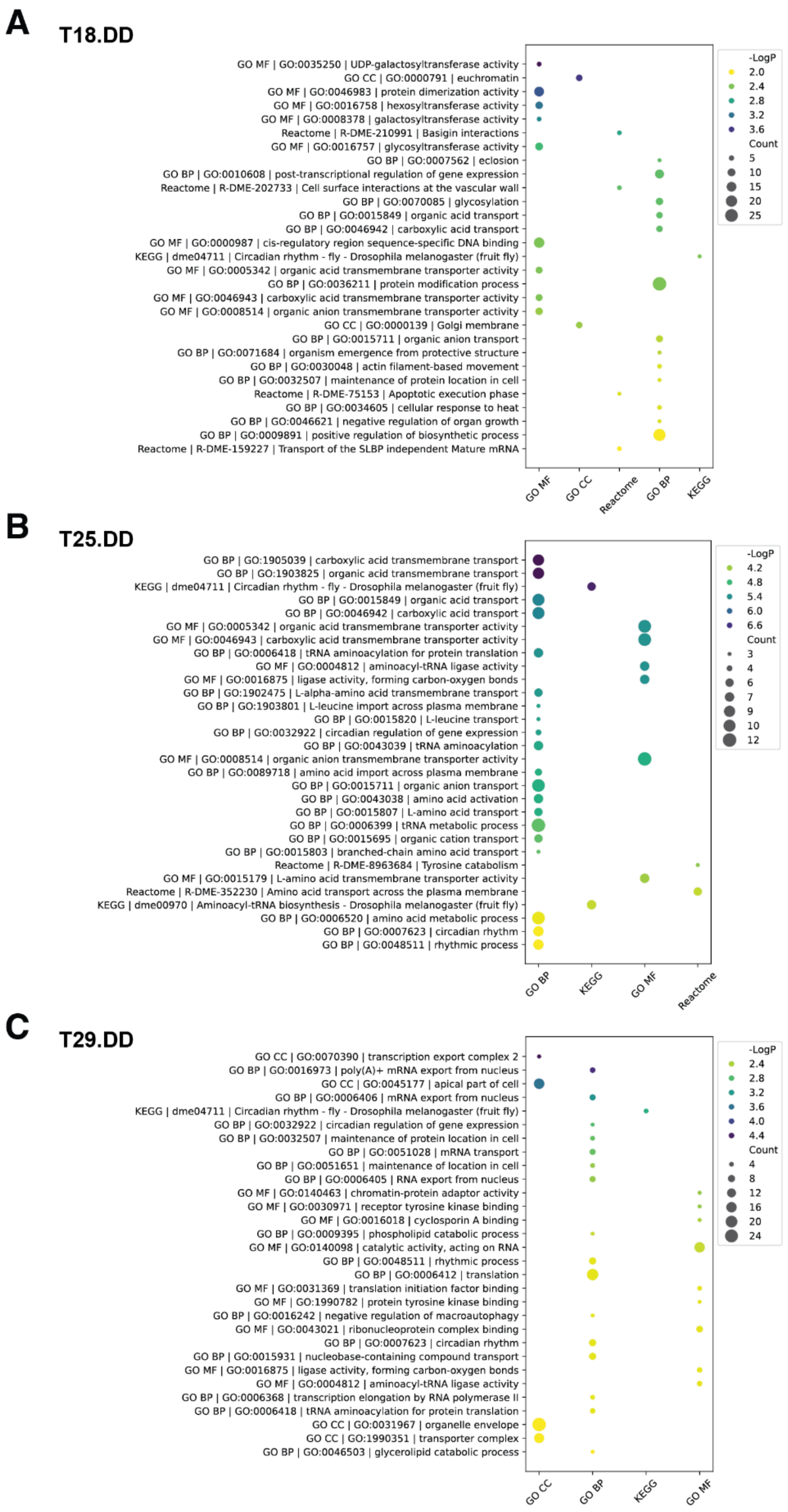

bioRxiv preprint doi: https://doi.org/10.1101/2025.11.04.686609; this version posted November 4, 2025. The copyright holder has placed this preprint (which was not certified by peer review) in the Public Domain. It is no longer restricted by copyright. Anyone can legally share, reuse, remix, or adapt this material for any purpose without crediting the original authors.

Figure S5. Gene ontology enrichment analysis for oscillating genes under each of the constant darkness conditions.

(A-C) Oscillating genes under 18°C DD (A), 25°C DD (B), and 29°C DD (C) conditions were analyzed for enrichment using Gene Ontology (GO), KEGG Pathway, and Reactome Gene Sets. The identified enriched terms were plotted based on their *p*-value and count.

bioRxiv preprint doi: https://doi.org/10.1101/2025.11.04.686609; this version posted November 4, 2025. The copyright holder has placed this preprint (which was not certified by peer review) in the Public Domain. It is no longer restricted by copyright. Anyone can legally share, reuse, remix, or adapt this material for any purpose without crediting the original authors.

Figure S6

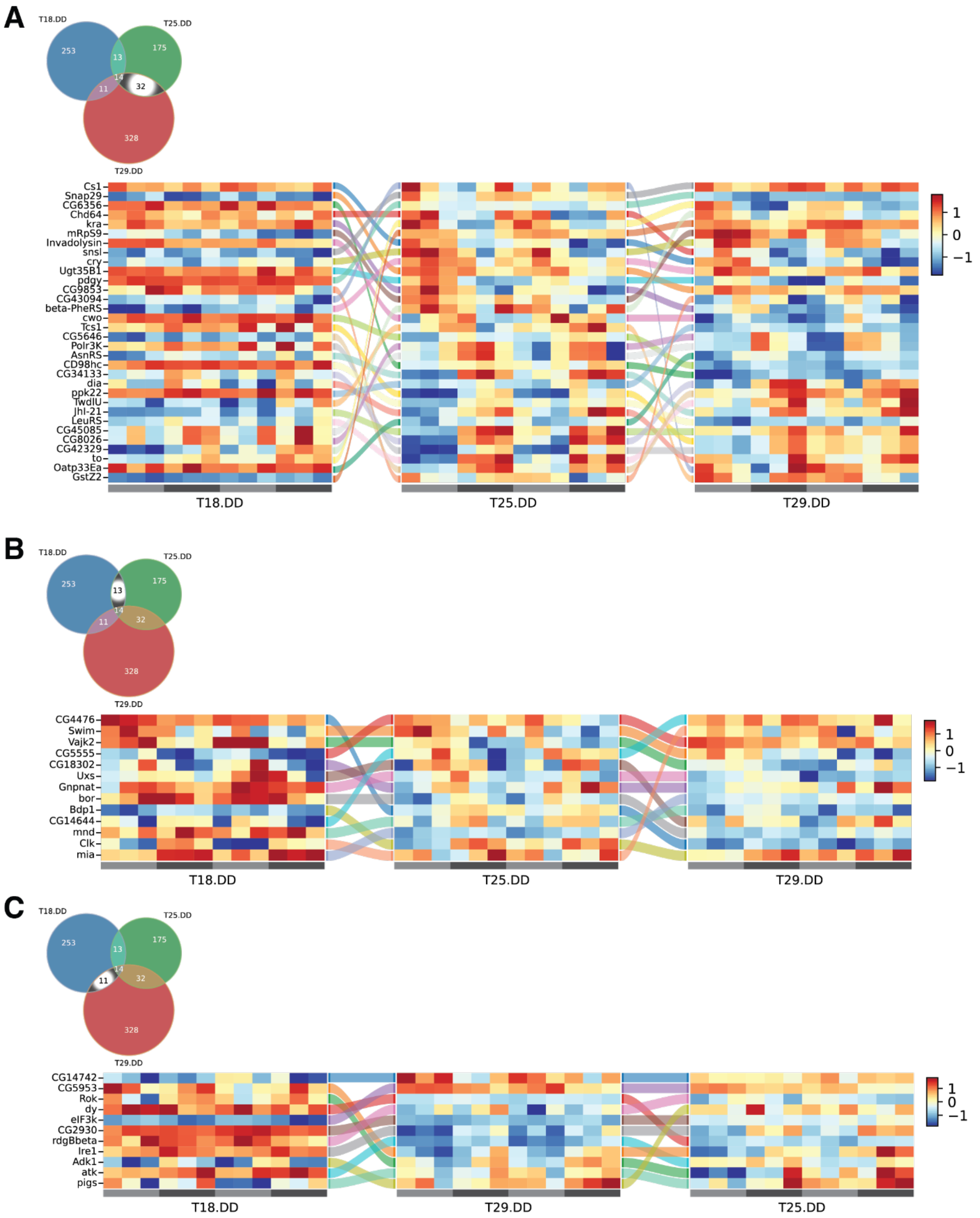

Figure S6. Shared oscillating genes under two of the three constant darkness conditions.

bioRxiv preprint doi: https://doi.org/10.1101/2025.11.04.686609; this version posted November 4, 2025. The copyright holder has placed this preprint (which was not certified by peer review) in the Public Domain. It is no longer restricted by copyright. Anyone can legally share, reuse, remix, or adapt this material for any purpose without crediting the original authors.

(A) 32 genes were identified as shared oscillating genes under 25°C DD and 29°C DD conditions, but not under the 18°C DD condition.

(B) 13 genes were identified as shared oscillating genes under 18°C DD and 25°C DD conditions, but not under the 29 °C DD condition.

(C) 11 genes were identified as shared oscillating genes under 18°C DD and 29°C DD conditions, but not under the 25°C DD condition.

(A-C) The Sankey diagrams illustrate how the oscillatory properties of these genes (A, 32 genes; B, 13 genes; C, 11 genes) change relative to each other. In each temperature-specific heatmap, genes are ordered by phase, and identical genes are connected across conditions by flow lines.
